# A Symptomatic De Novo Pheochromocytoma 23 Years after Liver Transplantation: A Case Report and Review of the Literature

**DOI:** 10.1155/2014/934385

**Published:** 2014-12-14

**Authors:** M. I. Montenovo, F. G. Jalikis, B. Hoch, R. Bakthavatsalam

**Affiliations:** ^1^Division of Transplantation, Department of Surgery, University of Washington, Seattle, WA, USA; ^2^Department of Anatomic Pathology, University of Washington, Seattle, WA, USA

## Abstract

We report a case of subacute onset of headaches and tremors with a newly discovered adrenal pheochromocytoma 23 years after an orthotopic liver transplantation and provide a review of the scarce literature regarding endocrine malignancies in liver transplant recipients. We describe the clinical presentation, diagnostic work-up, and management. This is the second case report in the literature of a de novo pheochromocytoma after solid organ transplantation. It shows that new-onset common symptoms in transplant recipients are always challenging and deserve a very thorough work-up until the cause of the symptoms is elucidated. A broad differential diagnosis should always be included in the study of any abnormalities in this patient population.

## 1. Introduction

Headaches are a very common symptom in adult liver transplant recipients. Immunosuppressant medications must always be considered a possible etiology [1] when assessing the origins of this symptom in transplant patients.

In addition to having an increased risk of developing migraines, malignancy in recipients has emerged as a prevailing cause of late morbidity and mortality [2]. Skin cancer and posttransplant lymphoproliferative disease comprise the most common malignancies after liver transplantation, followed by other solid organ cancers. Endocrine tumors are exceedingly rare in this population. Only one case of de novo pheochromocytoma has been previously reported in a liver transplant recipient who presented with diabetes mellitus [3].

Classically, pheochromocytomas present with episodic headache, HTN, palpitations, and sweating.

In this case report we describe a patient who was diagnosed with pheochromocytoma in association with new onset of migraines 23 years after liver transplantation.

## 2. Case Report

**Figure 1:**
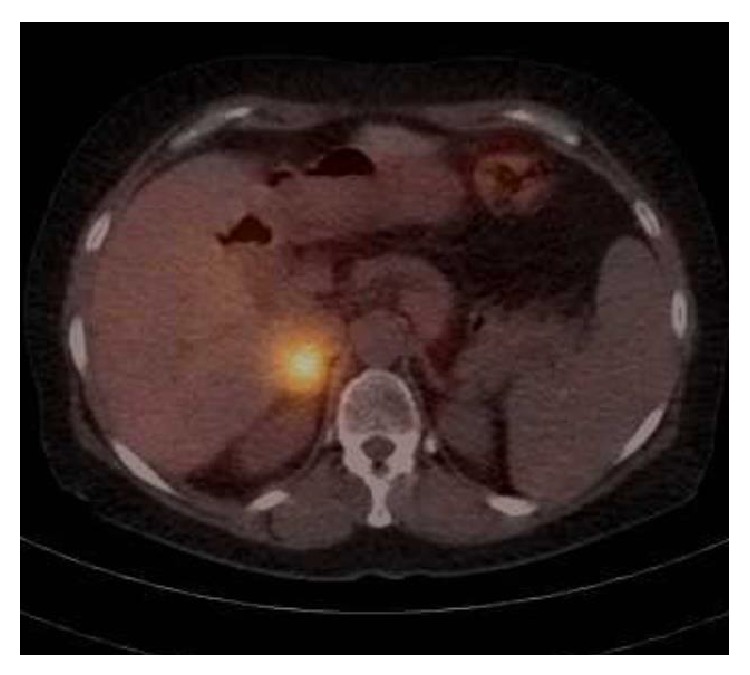
MIBG scan shows avid uptake of radiotracer in the right adrenal gland.

**Figure 2:**
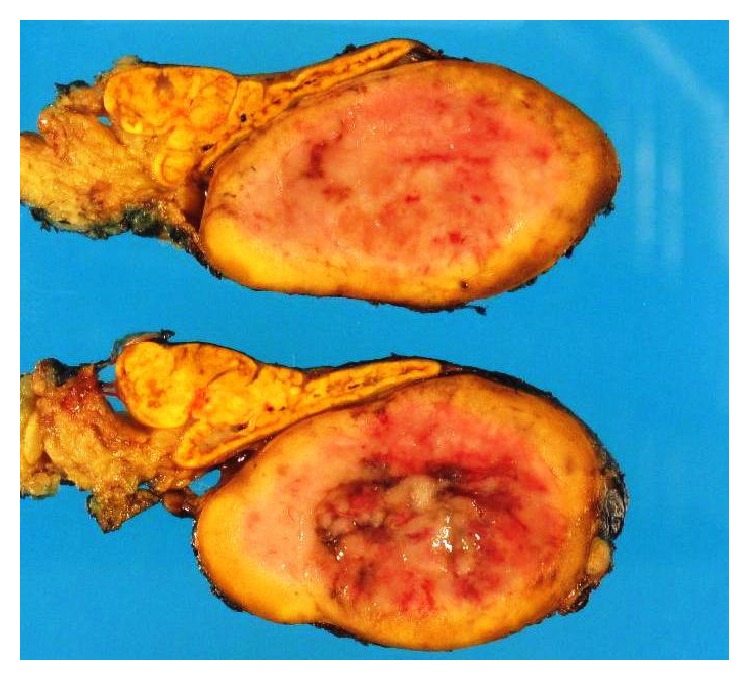
Gross appearance of the pheochromocytoma specimen.

**Figure 3:**
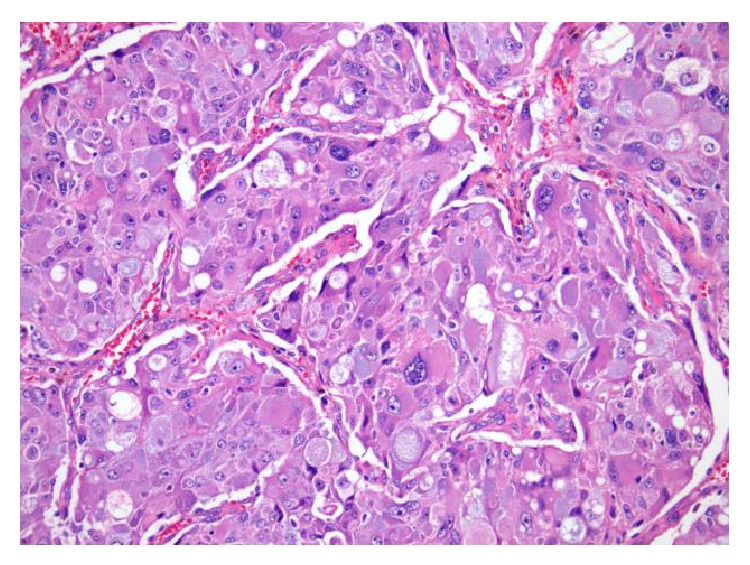
Histological examination revealed a neoplasm characterized by variably atypical cells exhibiting different growth patterns. Depicted here are pleomorphic neoplastic cells with intracytoplasmic hyaline globules (H&E stain, 20x).

A 61-year-old lady with a history of end-stage liver disease secondary to autoimmune hepatitis after orthotopic liver transplantation in 1990 presented at her annual check-up clinic with new-onset episodic tremors and debilitating migraines. She had a previous history of tremors immediately after the transplant which disappeared after several months. Over the last year she has developed recurrent episodic tremors which have significantly progressed in the last few months. She also developed a new onset of episodic severe headaches without aura for which she was started on sumatriptan with only partial relief. She denied any shortness of breath, palpitations, or hypertension. There was no other significant medical history. She denies any family history concerning pheochromocytoma or multiple endocrine neoplasia syndrome. For immunosuppression, she was on cyclosporine 100 mg QD, azathioprine 50 mg QD, and prednisone 1 mg QD. Her routine annual abdominal ultrasound protocol showed a new incidental heterogeneous mass measuring 3.1 cm × 2.2 cm × 2.6 cm in the right adrenal gland. A computed tomography with intravenous contrast demonstrated a 2.9 cm × 2.8 cm indeterminate nodule in the right adrenal mass of intermediate density (42 HU). A 24-hour urine collection demonstrated total urine metanephrines of 2,973 mcg/24 hr (reference <616 mcg/24 hr) and urine normetanephrine of 2,304 mcg/24 hr (reference <521 mcg/24 hr). An MIBG (iodine-123-meta-iodobenzylguanidine) scan showed avid uptake of radiotracer in the right adrenal gland with otherwise normal physiological distribution (Figure 1). The patient was started on phenoxybenzamine 10 mg BID ten days before the operation and titrated to orthostatic symptoms. She was admitted the day before the operation for appropriate hydration and underwent an uneventful open right adrenalectomy. She was discharged home on postoperative day 5, free of tremors and headaches. The final pathology report showed a pheochromocytoma (Figures 2 and 3).

She has remained asymptomatic and off antimigraine medication since discharge.

## 3. Discussion

Improved posttransplant patient and graft survival has been associated with an increased prevalence and incidence of posttransplant malignancies. Malignancies are a major cause of late death in liver transplant recipients [2]. The increased risk of cancer associated with duration and intensity of immunosuppression in organ transplant recipients [4] is well recognized. There is evidence of an increased incidence of skin, cervical, and lymphoid tumors after liver transplantation [5]. Endocrine tumors have rarely been reported in the medical literature. These case reports include a glucagonoma in a kidney transplant recipient, small cell neuroendocrine tumors of the small intestine in kidney, liver, and heart transplant recipients [6–8], and a pheochromocytoma in a liver transplant recipient [3].

In addition to the increased risk of tumors, transplant recipients also have an increased risk of new onset or exacerbation of headaches. Mild to severe headaches are a very common symptom in adult liver transplant recipients. Several conditions can lead to headaches and warrant special consideration. The neurotoxicity of immunosuppressants is commonly manifested as headaches [1]. Often, immunosuppressant-associated headaches are migraine-like and may occur de novo, or preexisting migraines may be exacerbated or may recur with immunosuppressant introduction. Patients susceptible to migraines experience increased severity and frequency of headaches when using calcineurin inhibitors, particularly tacrolimus. The incidence of headaches in patients treated with tacrolimus is 32% versus 21% with cyclosporine use [9]. Whereas cyclosporine neurotoxicity does not directly correlate with blood levels, the risk of tacrolimus-associated headache is significantly correlated with the tacrolimus blood level. Headaches can also be a manifestation of hypertension and, as such, can herald impending cerebrovascular accidents. Meningitis is always a concern in immunosuppressed patients and should be part of the differential diagnosis of headaches.

Pheochromocytomas are catecholamine-secreting tumors that arise from chromaffin cells of the adrenal medulla. They are rare tumors, with an estimated annual incidence of 0.8 per 100,000 person years [10]. The classic triad of symptoms in patients with pheochromocytoma consists of episodic headache, sweating, and tachycardia. About half have paroxysmal hypertension. The symptoms are caused by tumoral hypersecretion of norepinephrine, epinephrine, and dopamine. Approximately 95% of pheochromocytomas are in the abdomen, 85% to 90% of which are intra-adrenal. About 10% are malignant. Malignant pheochromocytomas are histologically and biochemically the same as benign ones. The only diagnostic tool of a malignant pheochromocytoma is local invasion into surrounding tissues and organs or distant metastases, which may occur up to 20 years after resection [11].

In summary, this is the second case report in the literature of a de novo pheochromocytoma after solid organ transplantation. It shows that new onset of common symptoms in transplant recipients is always challenging and deserves a very thorough work-up until the cause of the symptoms is elucidated. A broad differential diagnosis should always be included in the study of any abnormalities in this patient population. The case highlights as well the significant disability that a pheochromocytoma can cause and the complete resolution of the symptoms after removal of the tumor.
